# The Latest Fad

**Published:** 1891-02

**Authors:** 


### THE LATEST FAD.

Fads and fashions are ever changing, and what to-day is "all the rage" to-morrow finds itself supplanted by a new rival, and the old "fad" becomes a thing of ridicule and detestation. Fads in dress, fads in the mannerisms of society and fads in slang are expected, and a suffering public has learned to endure them as they come, though with what symptoms of nausea perhaps is never known. But what can we think when a disease which is often terrible in its effects becomes the popular fad, and every poor unfortunate who gets "a bad cold" immediately declares himself in the clutches of "The Grip," and dying, perhaps finds it less bitter than if he had fallen a victim to a more prosaic and less fashionable disease. Should "he live to tell the tale" with what feelings of pleasure does he relate to his friends how he "had the grip and like to a-died." "Anti-fat" doctors have lately reaped a harvest from the fashionable desire of reducing the bodily weight, and so we suspect has this "grip fad" reduced the weight of many a patient's purse and increased the weight of that of many a hard-worked and deserving doctor.

We hold "La Grippe" in deepest respect and awe, and pity those who fall in his grasp, but let us hope that the newspapers shall soon announce that "the grip" is gone, and that the public may now resume their old, winter ailment, the common, every-day "bad cold."

